# Supplementary material for: Linear Radical Additions-Coupling Polymerization (LRAsCP): Model, Experiment and Application
Source: Polymers (Basel). 2025 Mar 12;17(6):741. doi: 10.3390/polym17060741 (PMC11945175; doi:10.3390/polym17060741)
Supplement: Supplementary file 1 [file polymers-17-00741-s001.zip › polymers-3499047-supplementary.pdf]

Electronic Supplementary Information

for

Linear Radical Additions-Coupling Polymerization (LRAsCP): Model, Experiment and Application

Yudian Jiang[1], Kun Cao[2], Qi Wang[1,*]

[1]MOE Key Laboratory of Macromolecular Synthesis and Functionalization, Department of Polymer Science & Engineering, Zhejiang University, Hangzhou, 310058, P. R. China.

[2]State Key Laboratory of Chemical Engineering, College of Chemical and Biological Engineering, Zhejiang University, Hangzhou 310058, P. R. China.

* Corresponding author, wangq@zju.edu.cn

Outline

1. Theoretical analysis of LRAsCP P.3
2. Experimental Section P.15
  2.1. Materials and methods P.15
  2.2 Characterization P.15
  2.3 Synthesis of initiator P.15
  2.4 Polymerization procedure P.16
3. Tables P.19
4. Collection of spectra P.24
  4.1 NMR spectra of initiators and MBcPs P.24
  4.2 GPC curves of polymers P.27

1 Theoretical analysis of LRAsCP

1.1General process of LRAsCP

The process of LRAsCP is shown in **Scheme 2**. It includes three steps, such as initiation, propagation and coupling/termination, similar as normal radical polymerization. In the initiation step, bifunctional initiator (BFI), I_1XI_2, generates three kinds of radicals , such as I_2XI_1•, I_1XI_2• and •I_1XI_2•. The first two radicals are monoradical, and the third one is biradical. In the propagation step, three small radicals generate three macroradicals through radical addition reaction with monomer A, such as I_2XA_j•, I_1XA_j• and •A_kXA_j•. The number of macroradicals are the same as corresponding small radicals. In the coupling/termination step, radical coupling reaction between two radicals is a chain extension step instead of termination step. Other reactions, such as disproportionation and chain transfer reaction, are termination steps which result in a dead chain end of unit A. If the radical is considered as a functional group, the coupling reactions between macroradicals are a kind of coupling polymerization.

Let q_1 and q_2 be the extent of initiation of groups I_1 and I_2 of BFI, q be the total the extent of initiation of BFI, then q is equal to $0.5(q_1+q_2)$. Let ϕ_A be the termination factor involving radical A, which is the ratio of the rate of coupling reaction (R_c) to overall rate of reaction consuming radicals (R_t), R_c/R_t.

1.1.1 Initiation

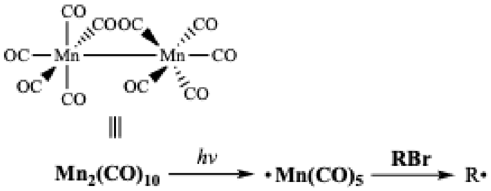

Scheme S1 Initiation reaction of visible-light induced decomposition of decacarbonyl dimanganese ($Mn_2(CO)_{10}$)

In the initiation step, the carbon radical was formed by radical transfer reaction between bromides and pentacarbonyl manganese radical ($Mn(CO)_5$•) generated by homolysis photolysis of decacarbonyl dimanganese ($Mn_2(CO)_{10}$) under visible light. (**Scheme S1**). Three small radicals are formed when BFI is used, which is shown in **Scheme 1**. Let δ_{11}, δ_{12}, δ_2 and δ_0 be the molar fraction

of small radicals $I_2XI_1\bullet$, $I_1XI_2\bullet$, $\bullet I_1XI_2\bullet$ and remaining BFI. Four molar fractions are given by Eqn. s1.

$$\delta_{11} = \overline{q_2}q_1 \tag{s1a}$$

$$\delta_{12} = \overline{q_1}q_2 \tag{s1b}$$

$$\delta_2 = q_1q_2 \tag{s1c}$$

$$\delta_0 = \overline{q_1q_2} \tag{s1d}$$

$$\overline{q_1} = 1 - q_1 \ \ , \ \overline{q_2} = 1 - q_2 \tag{s1e}$$

$$\sum \delta = 1 \tag{s1f}$$

1.1.2 Propagation

The macroradicals, $I_2XA_j\bullet$, $I_1XA_j\bullet$ and $\bullet A_kXA_j\bullet$, are formed via radical addition reaction of the corresponding small radicals to monomer A. Their molar fractions are equals to δ_{11}, δ_{12} and δ_2, in terms of molecule, respectively. In terms of the radicals, the total number of radicals is (δ_{11}+δ_{12}+2δ_2) if the initial number of BFI is unity. The molar fractions of three macroradicals (θ) are given by Eqn. s2.

$$\theta_{11} = \frac{\delta_{11}}{\delta_{11}+\delta_{12}+2\delta_2} = \frac{q_1\overline{q_2}}{q_1+q_2} \tag{s2a}$$

$$\theta_{12} = \frac{\delta_{12}}{\delta_{11}+\delta_{12}+2\delta_2} = \frac{q_2\overline{q_1}}{q_1+q_2} \tag{s2b}$$

$$\theta_2 = \frac{2\delta_2}{\delta_{11}+\delta_{12}+2\delta_2} = \frac{2q_1q_2}{q_1+q_2} \tag{s2c}$$

$$\sum \theta = 1 \tag{s2d}$$

1.1.3 Coupling/termination

The coupling reaction between three macroradicals generates six multiblock polymers (MBP), which can be divided into three types. The first type is polymer having two terminal units A, denoted as AA. The second type is polymer having one functional group I_1 or I_2, denoted as I_1A and I_2A. Both are collectively denoted as IA. The third one is polymer having two functional groups I_1 or I_2, denoted as I_1AI_1, I_1AI_2 and I_2AI_2. All three are collectively denoted as IAI.

1.2 Probability of coupling reaction

If the termination factor is ϕ_A, the total probability of radical undergoing non-coupling reaction is $1-\phi_A$ or $\overline{\phi_A}$. The probability of one radical coupled with macroradicals, such as I_2XA_j•, I_1XA_j• and •A_kXA_j•, is $\theta_{11}\phi_A$, $\theta_{12}\phi_A$ and $\theta_2\phi_A$ respectively.

1.3 Number-distribution function of various MBPs ($p_{n,MBP}$)

Based on the assumption of equal reactivity of all radicals, the number-distribution functions of six MBPs are given by

$$p_{AA,n} = \delta_2(\theta_2\phi_A)^{n-1}\overline{\phi_A}^2 = q_1q_2\overline{\phi_A}^2(\frac{2q_1q_2}{q_1+q_2}\phi_A)^{n-1} \quad (n \geq 1) \qquad \text{(s3a)}$$

$$p_{I_1A,n} = \delta_{12}(\theta_2\phi_A)^{n-1}\overline{\phi_A} = \overline{q_1}q_2\overline{\phi_A}\left(\frac{2q_1q_2}{q_1+q_2}\phi_A\right)^{n-1} \quad (n \geq 1) \qquad \text{(s3b)}$$

$$p_{I_2A,n} = \delta_{11}(\theta_2\phi_A)^{n-1}\overline{\phi_A} = \overline{q_2}q_1\overline{\phi_A}\left(\frac{2q_1q_2}{q_1+q_2}\phi_A\right)^{n-1} \quad (n \geq 1) \qquad \text{(s3c)}$$

$$p_{I_1AI_1,n} = 0.5\delta_{12}(\theta_2\phi_A)^{n-2}\theta_{12}\phi_A = 0.5\frac{(q_2\overline{q_1})^2}{q_1+q_2}\phi_A\left(\frac{2q_1q_2}{q_1+q_2}\phi_A\right)^{n-2} \quad (n \geq 2) \qquad \text{(s3d)}$$

$$p_{I_1AI_2,n} = \delta_{12}(\theta_2\phi_A)^{n-2}\theta_{11}\phi_A = \frac{q_1q_2\overline{q_1q_2}}{q_1+q_2}\phi_A\left(\frac{2q_1q_2}{q_1+q_2}\phi_A\right)^{n-2} \quad (n \geq 2) \qquad \text{(s3e)}$$

$$p_{I_2AI_2,n} = 0.5\delta_{11}(\theta_2\phi_A)^{n-2}\theta_{11}\phi_A = 0.5\frac{(q_1\overline{q_2})^2}{q_1+q_2}\phi_A\left(\frac{2q_1q_2}{q_1+q_2}\phi_A\right)^{n-2} \quad (n \geq 2) \qquad \text{(s3f)}$$

where n is the number of X, residual moiety of BFIs.

For example, coupling of n biradical •A_kXA_j• results in the formation of AA-type of MBP containing n BFI units. The formation of multiblock structure starts with one •A_kXA_j•, followed by (n-1) coupling reaction of the same biradical, and terminated with 2 non-coupling reactions of two terminal radicals. The corresponding probabilities for three steps are δ_2, $(\theta_2\phi_A)^{n-1}$ and $\overline{\phi_A}^2$. The product of three probabilities yields Eqn. 3a. The formation of I_1AI_2-type MBP starts with one I_1XI_2•, followed by coupling reaction of (n-2) •A_kXA_j•, and terminated by coupling with one I_2XI_1•. The product of δ_{12}, $(\theta_2\phi_A)^{n-2}$ and $\theta_{11}\phi_A$ yields Eqn. s3e. The factor of 0.5 in Eqn. s3d and s3f is due to the symmetry of I_1AI_1- and I_2AI_2-type MBPs.

1.4 Numbers/Fractions of various MBPs

1.4.1 The number of various MBPs (N_{MBP})

Let the initial number of BFI be unity, summation of Eqn. s3 yields the number of six MBPs, which are

$$N_{AA}=\sum_{n=1}p_{AA,n}=\frac{\delta_2\overline{\phi_A}^2}{1-\theta_2\phi_A}=q_1q_2\overline{\phi_A}^2\frac{q_1+q_2}{q_1+q_2-2q_1q_2\phi_A} \tag{s4a}$$

$$N_{I_1A}=\sum_{n=1}p_{I_1A,n}=\frac{\delta_{11}\overline{\phi_A}}{1-\theta_2\phi_A}=\overline{q_1}q_2\overline{\phi_A}\frac{q_1+q_2}{q_1+q_2-2q_1q_2\phi_A} \tag{s4b}$$

$$N_{I_2A}=\sum_{n=1}p_{I_2A,n}=\frac{\delta_{12}\overline{\phi_A}}{1-\theta_2\phi_A}=\overline{q_2}q_1\overline{\phi_A}\frac{q_1+q_2}{q_1+q_2-2q_1q_2\phi_A} \tag{s4c}$$

$$N_{I_1AI_1}=\sum_{n=2}p_{I_1AI_1,n}=0.5\frac{\delta_{11}\theta_{11}\phi_A}{1-\theta_2\phi_A}=0.5\frac{(q_2\overline{q_1})^2}{q_1+q_2}\phi_A\frac{q_1+q_2}{q_1+q_2-2q_1q_2\phi_A} \tag{s4d}$$

$$N_{I_1AI_2}=\sum_{n=2}p_{I_1AI_2,n}=\frac{\delta_{11}\theta_{12}\phi_A}{1-\theta_2\phi_A}=\frac{q_1q_2\overline{q_1}\,\overline{q_2}}{q_1+q_2}\phi_A\frac{q_1+q_2}{q_1+q_2-2q_1q_2\phi_A} \tag{s4e}$$

$$N_{I_2AI_2}=\sum_{n=2}p_{I_2AI_2,n}=0.5\frac{\delta_{12}\theta_{12}\phi_A}{1-\theta_2\phi_A}=0.5\frac{(q_1\overline{q_2})^2}{q_1+q_2}\phi_A\frac{q_1+q_2}{q_1+q_2-2q_1q_2\phi_A} \tag{s4f}$$

Combining Eqns. s4b and s4c yields

$$N_{IA}=N_{I_1A}+N_{I_2A}=\frac{(q_1+q_2)(q_1+q_2-2q_1q_2)\overline{\phi_A}}{q_1+q_2-2q_1q_2\phi_A} \tag{s4g}$$

Combining Eqns. s4d ~ s4f yields

$$N_{IAI}=N_{I_1AI_1}+N_{I_1AI_2}+N_{I_2AI_2}=\frac{0.5(q_1+q_2-2q_1q_2)^2\phi_A}{q_1+q_2-2q_1q_2\phi_A} \tag{s4h}$$

Combining Eqns. s4a, s4g and s4h yields the total number of MBPs (N_{P_A}).

$$N_{P_A}=N_{AA}+N_{IA}+N_{IAI}=(q_1+q_2)(1-0.5\phi_A)-q_1q_2 \tag{s4i}$$

1.4.2 Molar/weight fractions of various MBPs (F_{MBP}/W_{MBP})

The molar fractions of three types of MBPs can be calculated by

$$F_{AA}=\frac{N_{AA}}{N_{P_A}}=\frac{q_1q_2(q_1+q_2)(1-\phi_A)^2}{(q_1+q_2-2q_1q_2\phi_A)[(q_1+q_2)(1-0.5\phi_A)-q_1q_2]} \tag{s5a}$$

$$F_{IA}=\frac{N_{IA}}{N_{P_A}}=\frac{(q_1+q_2)(q_1+q_2-2q_1q_2)\overline{\phi_A}}{(q_1+q_2-2q_1q_2\phi_A)((q_1+q_2)(1-0.5\phi_A)-q_1q_2)} \tag{s5b}$$

$$F_{IAI}=\frac{N_{IAI}}{N_{P_A}}=\frac{0.5\phi_A(q_1+q_2-2q_1q_2)^2}{(q_1+q_2-2q_1q_2\phi_A)((q_1+q_2)(1-0.5\phi_A)-q_1q_2)} \tag{s5c}$$

The weight fraction of AA-type MBP in terms of X can be calculated by

$$W_{AA}=\sum_{n=1}np_{AA,n}=q_1q_2\overline{\phi_A}^2\sum n\left(\frac{2q_1q_2}{q_1+q_2}\phi_A\right)^{n-1}=q_1q_2\overline{\phi_A}^2\left(\frac{q_1+q_2}{q_1+q_2-2q_1q_2\phi_A}\right)^2 \tag{s6}$$

1.5 Number-average degree of polymerization of various MBPs ($DP_{\mathrm{n,MBP}}$)

The number-average degree of polymerization (in terms of X) of six MBPs, DP_{n}, can be derived from the number-distribution function, respectively. Six DP_{n} are given by

$$DP_{n,AA}=\frac{\sum_{n=1} np_{AA,n}}{\sum_{n=1} p_{AA,n}}=\frac{\sum_{n=1} n(\theta_2\phi_A)^{n-1}}{\sum_{n=1}(\theta_2\phi_A)^{n-1}}=\frac{1}{1-\theta_2\phi_A}=\frac{q_1+q_2}{q_1+q_2-2q_1q_2\phi_A} \quad \text{(s7a)}$$

$$DP_{n,I_1A}=\frac{\sum_{n=1} np_{I_1A,n}}{\sum_{n=1} p_{I_1A,n}}=\frac{\sum_{n=1} n(\theta_2\phi_A)^{n-1}}{\sum_{n=1}(\theta_2\phi_A)^{n-1}}=\frac{1}{1-\theta_2\phi_A}=\frac{q_1+q_2}{q_1+q_2-2q_1q_2\phi_A} \quad \text{(s7b)}$$

$$DP_{n,I_2A}=\frac{\sum_{n=1} np_{I_2A,n}}{\sum_{n=1} p_{I_2A,n}}=\frac{\sum_{n=1} n(\theta_2\phi_A)^{n-1}}{\sum_{n=1}(\theta_2\phi_A)^{n-1}}=\frac{1}{1-\theta_2\phi_A}=\frac{q_1+q_2}{q_1+q_2-2q_1q_2\phi_A} \quad \text{(s7c)}$$

$$DP_{n,I_1AI_1}=\frac{\sum_{n=2} np_{I_1AI_1,n}}{\sum_{n=2} p_{I_1AI_1,n}}=\frac{\sum_{n=2} n(\theta_2\phi_A)^{n-2}}{\sum_{n=2}(\theta_2\phi_A)^{n-2}}=\frac{2-\theta_2\phi_A}{1-\theta_2\phi_A}=2\frac{q_1+q_2-q_1q_2\phi_A}{q_1+q_2-2q_1q_2\phi_A} \quad \text{(s7d)}$$

$$DP_{n,I_1AI_2}=\frac{\sum_{n=2} np_{I_1AI_2,n}}{\sum_{n=2} p_{I_1AI_2,n}}=\frac{\sum_{n=2} n(\theta_2\phi_A)^{n-2}}{\sum_{n=2}(\theta_2\phi_A)^{n-2}}=\frac{2-\theta_2\phi_A}{1-\theta_2\phi_A}=2\frac{q_1+q_2-q_1q_2\phi_A}{q_1+q_2-2q_1q_2\phi_A} \quad \text{(s7e)}$$

$$DP_{n,I_2AI_2}=\frac{\sum_{n=2} np_{I_2AI_2,n}}{\sum_{n=2} p_{I_2AI_2,n}}=\frac{\sum_{n=2} n(\theta_2\phi_A)^{n-2}}{\sum_{n=2}(\theta_2\phi_A)^{n-2}}=\frac{2-\theta_2\phi_A}{1-\theta_2\phi_A}=2\frac{q_1+q_2-q_1q_2\phi_A}{q_1+q_2-2q_1q_2\phi_A} \quad \text{(s7f)}$$

Combining Eqns. s7b and s7c yields

$$DP_{n,IA}=\frac{q_1+q_2}{q_1+q_2-2q_1q_2\phi_A} \quad \text{(s7g)}$$

Combining Eqns. s7d ~ s7f yields

$$DP_{n,IAI}=2\frac{q_1+q_2-q_1q_2\phi_A}{q_1+q_2-2q_1q_2\phi_A}=DP_{n,IA}+1 \quad \text{(s7h)}$$

1.6 The number-average degree of polymerization (*DP*$_n$), the number of blocks per MBP (*N*$_K$) and number-average molar mass (*M*$_n$) of total MBP

1.6.1 Total number of MBPs (N_{P_A})

The total number of MBPs can be deduced by another way. The number of terminal group A (N_A) generated by non-coupling reactions of radicals is given by the product of sum of radical and probability of non-coupling reaction, which is

$$N_A=(q_1+q_2)\overline{\phi_A} \quad \text{(s8a)}$$

The number of terminal groups I_1 and I_2 incorporated into the MBPs (N_I) is given by

$$N_I=\overline{q_1}+\overline{q_2}-2\overline{q_1q_2}=q_1+q_2-2q_1q_2 \quad \text{(s8b)}$$

$$N_{P_A}=0.5(N_A+N_I)=(q_1+q_2)(1-0.5\phi_A)-q_1q_2 \quad \text{(s8c)}$$

Since the number of linear polymers is half of the number of terminal groups, the total number of MBPs (N_{P_A}) is given by Eqn. s8c, which is the same as Eqn. s4i derived from distribution functions. This result also confirms the validity of the distribution function.

1.6.2 *DP*$_n$ of total MBPs

The number of X incorporated into the MBPs (N_X) is given by

$$N_X = 1 - \overline{q_1 q_2} = q_1 + q_2 - q_1 q_2 \quad \text{(s9a)}$$

Therefore, the DP_n of total MBPs in terms of X is given by

$$DP_n = \frac{N_X}{N_{P_A}} = \frac{q_1+q_2-q_1q_2}{(q_1+q_2)(1-0.5\phi_A)-q_1q_2} \quad \text{(s9b)}$$

1.6.3 N_K of total MBPs

The total number of polymer blocks formed in the polymerization is given by

$$(q_1 + q_2)(1 - 0.5\phi_A) \quad \text{(s10a)}$$

The average number of blocks per MBP (N_K) can be is given by

$$N_K = \frac{(q_1+q_2)(1-0.5\phi_A)}{N_{P_A}} = \frac{(q_1+q_2)(1-0.5\phi_A)}{(q_1+q_2)(1-0.5\phi_A)-q_1q_2} \quad \text{(s10b)}$$

1.6.4 M_n of total MBPs

The number-average molar mass of total MBPs (M_n) is

$$M_n = \frac{A_0 C m_A}{X_0 N_{P_A}} = \frac{A_0 C m_A}{((q_1+q_2)(1-0.5\phi_A)-q_1q_2)X_0} \quad \text{(s11a)}$$

where A_0 and X_0 are initial concentration of monomer A and BFI, C is the conversion of monomer and m_A is the molar mass of monomer A. The ratio, A_0Cm_A/X_0, in Eqn. s11a is the molar mass of polymer upon each BFI generating one chain. The ratio is defined as apparent number-average molar mass ($M_{n,0}$) and is given by Eqn. s11b. Combination of Eqn. s11a and s11b yields Eqn. s11c.

$$M_{n,0} = \frac{A_0 C m_A}{X_0} \quad \text{(s11b)}$$

$$M_n = \frac{M_{n,0}}{(q_1+q_2)(1-0.5\phi_A)-q_1q_2} \quad \text{(s11c)}$$

1.7 Relationship between DP_n, N_K and M_n

In radical polymerization initiated by monofunctional initiator (MFI), the relationship between the kinetic chain length (v) and its number-average molar mass (M'_n) is given by Eqn. s12a

$$M'_n = \frac{v}{1-0.5\phi_A} \quad \text{(s12a)}$$

$$v = (1 - 0.5\phi_A)M'_n \quad \text{(s12b)}$$

So, v in the traditional radical polymerization can be estimated by ϕ and M'_n according to Eqn. s12b.

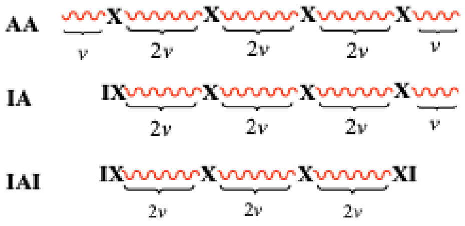

Scheme S2 The quantitively relationship between DP_n and $N_ν$ for various MBPs

As shown in **Scheme S2**, the relationship between the number of X (DP_n) and the number of kinetic chain ($N_ν$) per MBP depends on its type and is given by Eqn. s13a – s13c.

$$N_{\nu,AA} = 2DP_{n,AA} \quad \text{(s13a)}$$

$$N_{\nu,IA} = 2\left(DP_{n,IA} - 1\right) + 1 \quad \text{(s13b)}$$

$$N_{\nu,IAI} = 2\left(DP_{n,IAI} - 2\right) + 2 \quad \text{(s13c)}$$

Therefore, the overall relationship is

$$N_\nu = N_{\nu,AA}F_{AA} + N_{\nu,IA}F_{IA} + N_{\nu,IAI}F_{IAI} = 2\left(DP_{n,AA}F_{AA} + DP_{n,IA}F_{IA} + DP_{n,IAI}F_{IAI}\right) - (F_{IA} + 2F_{IAI}) = 2DP_n - (F_{IA} + 2F_{IAI}) \quad \text{(s13d)}$$

or

$$DP_n = 0.5N_\nu + (0.5F_{IA} + F_{IAI}) \quad \text{(s13e)}$$

Based on the definition of $N_ν$, its value is given by Eqn. s14.

$$N_\nu = \frac{M_n}{\nu} \quad \text{(s14)}$$

Combination of Eqn. s13e and s14, we get

$$DP_n = \frac{M_n}{2\nu} + (0.5F_{IA} + F_{IAI}) \quad \text{(s13f)}$$

ν obtained under the same ratio of monomer to functional group is the same for BFI and MFI, so ν can be evaluated by Eqn. s12b. Combination of Eqn. s12b and s13f yields

$$DP_n = \frac{M_n}{(2-\phi_A)M_n'} + (0.5F_{IA} + F_{IAI}) \quad \text{(s13g)}$$

MFI produces monoblock polymers, while BFI produces MBPs. At the same polymerization condition, the average length of the block generated by two initiators is the same. Since both M_n and M_n' can be estimated by GPC measurement of polymers prepared by MFI and BFI, their ratio is the experimental estimation of N_K, which is denoted by $N_{K,GPC}$ in Eqn. s15.

$$N_{K,GPC} = \frac{M_{n,GPC}}{M'_{n,GPC}} \quad \text{(s15)}$$

Combination of Eqn. s13g and s15 yields

$$DP_{n,GPC} = \frac{N_{K,GPC}}{2-\phi_A} + (0.5F_{IA} + F_{IAI}) \quad \text{(s13h)}$$

F_{IA} and F_{IAI} can be predicted by Eqn. s5b and s5c, therefore Eqn. s13h can be used to estimate the DP_n of MBP by experiment, denoted as $DP_{n,GPC}$.

1.8 Functionality of MBP

Let f be the number of functional groups (I_1 or I_2) per MBP or functionality of MBP. According to Eqn. s8b and s8c, f is given by

$$f = \frac{N_I}{N_{P_A}} = \frac{q_1+q_2-2q_1q_2}{(q_1+q_2)(1-0.5\phi_A)-q_1q_2} \quad \text{(s16)}$$

1.9 Two special cases

1.9.1 *s*-Initiation using symmetrical BFI

(a) If I_1 and I_2 are equivalent, we get q_1=q_2=q. This is a simultaneous initiation case termed *s*-initiation of symmetrical BFI. The related parameters related to MBPs are summarized to Eqn. s17.

$$N_{AA} = \frac{q^2\overline{\phi_A}^2}{1-q\phi_A} \quad \text{(s17a)}$$

$$N_{IA} = \frac{2q\bar{q}\overline{\phi_A}}{1-q\phi_A} \quad \text{(s17b)}$$

$$N_{IAI} = \frac{q\bar{q}^2\phi_A}{1-q\phi_A} \quad \text{(s17c)}$$

$$N_{P_A} = q(2-\phi_A-q) \quad \text{(s17d)}$$

$$F_{AA} = \frac{q\overline{\phi_A}^2}{(1-q\phi_A)(2-\phi_A-q)} \quad \text{(s17e)}$$

$$F_{IA} = \frac{2(1-q)\overline{\phi_A}}{(1-q\phi_A)(2-\phi_A-q)} \quad \text{(s17f)}$$

$$F_{IAI} = \frac{(1-q)^2\phi_A}{(1-q\phi_A)(2-\phi_A-q)} \quad \text{(s17g)}$$

$$W_{AA} = (\frac{q}{1-q\phi_A})^2\overline{\phi_A}^2 \quad \text{(s17h)}$$

$$DP_{n,AA} = \frac{1}{1-q\phi_A} \quad \text{(s17i)}$$

$$DP_{n,IA} = \frac{1}{1-q\phi_A} \quad \text{(s17j)}$$

$$DP_{n,IAI} = \frac{2-q\phi_A}{1-q\phi_A} \quad \text{(s17k)}$$

$$DP_n = \frac{2-q}{2-\phi_A-q} \quad \text{(s17m)}$$

$$DP_{n,GPC} = \frac{N_{K,GPC}}{2-\phi_A} + \frac{1-q}{2-\phi_A-q} \quad \text{(s17n)}$$

$$M_n = \frac{M_{n,0}}{q(2-q-\phi_A)} \quad \text{(s17o)}$$

$$N_K = \frac{2-\phi_A}{2-\phi_A-q} \quad \text{(s17p)}$$

$$f = \frac{2(1-q)}{(2-\phi_A-q)} \quad \text{(s17q)}$$

(b) Estimation of q

For s-initiation case using symmetrical BFI, $q_1 = q_2 = q$. Let $R = \frac{M_{n,0}}{M_n}$, Eqn. s17o is converted to

$$q^2 - (2-\phi_A)q + R = 0 \quad \text{(s18a)}$$

$$q = \frac{(2-\phi_A) \pm \sqrt{(2-\phi_A)^2 - 4R}}{2} \quad \text{(s18b)}$$

Solving Eqn. s18a yields Eqn. s18b, which can be used to estimate of q by ϕ_A and R.

1.9.2 *c*-Initiation using asymmetrical BFI

If the activity of functional group I_1 is extremely greater than that of I_2, I_2 only participates in the polymerization after I_1 is consumed. This is a cascade initiation case termed *c*-initiation. The total extent of initiation, q, is given by

$$q = \begin{cases} 0.5q_1, & q \le 0.5,\ q_2 = 0 \\ 0.5(1+q_2), & q > 0.5,\ q_1 = 1 \end{cases} \quad \text{(s19)}$$

1.9.2.1 In the case of q ≤ 0.5

(a) When q ≤ 0.5, substituting $q_1 = 2q$ and $q_2 = 0$ into related equations yields Eqn. s20.

$$N_{AA} = 0 \quad \text{(s20a)}$$

$$N_{I_1A} = 0 \ ; \ N_{I_2A} = 2q\overline{\phi_A} \ ; \ N_{IA} = 2q\overline{\phi_A} \quad \text{(s20b)}$$

$$N_{I_1AI_1} = 0 \ ; \ N_{I_1AI_2} = 0 \ ; \ N_{I_2AI_2} = q\phi_A \ ; \ N_{IAI} = q\phi_A \quad \text{(s20c)}$$

$$N_{P_A} = q(2-\phi_A) \quad \text{(s20d)}$$

$$F_{AA} = 0 \quad \text{(s20e)}$$

$$F_{IA} = \frac{\overline{\phi_A}}{1-0.5\phi_A} \quad \text{(s20f)}$$

$$F_{IAI} = \frac{0.5\phi_A}{1-0.5\phi_A} \quad \text{(s20g)}$$

$$W_{AA} = 0 \quad \text{(s20h)}$$

$$DP_{n,I_2A} = 1 \quad ; \quad DP_{n,I_2AI_2} = 2 \quad \text{(s20i)}$$

$$DP_n = \frac{1}{1-0.5\phi_A} \quad \text{(s20j)}$$

$$DP_{n,GPC} = \frac{N_{K,GPC}}{2-\phi_A} + \frac{0.5}{1-0.5\phi_A} \quad \text{(s20k)}$$

$$M_n = \frac{M_{n,0}}{q(2-\phi_A)} \quad \text{(s20m)}$$

$$N_K = 1 \quad \text{(s20n)}$$

$$f = \frac{1}{1-0.5\phi_A} \quad \text{(s20o)}$$

(b) Estimation of q

When $q \leq 0.5$, q_1=2q and q_2=0, solving Eqn. s20m yields Eqn. s21, which can be used to estimate of q by ϕ_A and R.

$$q = \frac{M_{n,0}}{(2-\phi_A)M_n} = \frac{R}{(2-\phi_A)} \quad \text{(s21)}$$

1.9.2.2 In the case of q > 0.5

(a) When q > 0.5, substituting q_1 = 1 and q_2 =2q-1 into related equations yields Eqn. s22.

$$N_{AA} = \frac{q_2(1+q_2)\overline{\phi_A}^2}{1+q_2-2q_2\phi_A} = \frac{q(2q-1)\overline{\phi_A}^2}{q+\phi_A-2q\phi_A} \quad \text{(s22a)}$$

$$N_{I_1A} = 0 \quad ; \quad N_{I_2A} = \frac{\overline{q_2}(1+q_2)\overline{\phi_A}}{1+q_2-2q_2\phi_A} = \frac{2q(1-q)\overline{\phi_A}}{q+\phi_A-2q\phi_A} \quad \text{(s22b)}$$

$$N_{I_1AI_1} = N_{I_1AI_2} = 0 \quad ; \quad N_{I_2AI_2} = \frac{0.5(\overline{q_2})^2\phi_A}{1+q_2-2q_2\phi_A} = \frac{\bar{q}^2\phi_A}{q+\phi_A-2q\phi_A} \quad \text{(s22c)}$$

$$N_{P_A} = 1 - q\phi_A \quad \text{(s22d)}$$

$$F_{AA} = \frac{q_2(1+q_2)(1-\phi_A)^2}{(1+q_2-2q_2\phi_A)[(1+q_2)(1-0.5\phi_A)-q_2]} = \frac{q(2q-1)\overline{\phi_A}^2}{(q+\phi_A-2q\phi_A)(1-q\phi_A)} \quad \text{(s22e)}$$

$$F_{IA} = \frac{2q(1-q)\overline{\phi_A}}{(q+\phi_A-2q\phi_A)(1-q\phi_A)} \quad \text{(s22f)}$$

$$F_{IAI} = \frac{(1-q)^2\phi_A}{(q+\phi_A-2q\phi_A)(1-q\phi_A)} \quad \text{(s22g)}$$

$$W_{AA} = \frac{(2q-1)q^2\overline{\phi_A}^2}{(q+\phi_A-2q\phi_A)^2} \quad \text{(s22h)}$$

$$DP_{n,AA} = DP_{n,I_2A} = \frac{1+q_2}{1+q_2-2q_2\phi_A} = \frac{q}{q+\phi_A-2q\phi_A} \quad \text{(s22i)}$$

$$DP_{n,I_2AI_2} = 2\,\frac{1+q_2-q_2\phi_A}{1+q_2-2q_2\phi_A} = DP_{n,I_2S_A} + 1 \quad \text{(s22j)}$$

$$DP_n = \frac{1}{1-0.5(1+q_2)\phi_A} = \frac{1}{1-q\phi_A} \quad \text{(s22k)}$$

$$DP_{n,GPC} = \frac{N_{K,GPC}}{2-\phi_A} + \frac{1-q}{1-q\phi_A} \quad \text{(s22m)}$$

$$M_n = \frac{M_{n,0}}{1-q\phi_A} \quad \text{(s22n)}$$

$$N_K = \frac{q(2-\phi_A)}{1-q\phi_A} \quad \text{(s22o)}$$

$$f = \frac{2(1-q)}{1-q\phi_A} \quad \text{(s22p)}$$

(b) Estimation of q

When $q > 0.5$, solving Eqn. s22n yields Eqn. s23, which yields q when R and ϕ_A are available.

$$q = \frac{1-R}{\phi_A} \quad \text{(s23)}$$

1.10 The second-step LRAsCP

At the polymerization time when the extent of initiation of I_1 and I_2 are q_{10} and q_{20}, replacing monomer A by monomer B leads to the second LRAsCP. The second polymerization produces copolymer blocks, and the resultant product is the multiblock copolymer (MBcP). At the beginning of the second polymerization, the component existing in the polymerization mixture are BFI and three types of MBPs of monomer A. According to Eqn. s1d and s4a, s4g and s4h, the number of each component is given by

$$I_1XI_2 = \overline{q_{10}q_{20}} \quad \text{(s24a)}$$

$$N_{AA} = \frac{q_{10}q_{20}(q_{10}+q_{20})\overline{\phi_A}^2}{q_{10}+q_{20}-2q_{10}q_{20}\phi_A} \quad \text{(s24b)}$$

$$N_{IA} = \frac{(q_{10}+q_{20})(q_{10}+q_{20}-2q_{10}q_{20})\overline{\phi_A}}{q_{10}+q_{20}-2q_{10}q_{20}\phi_A} \quad \text{(s24c)}$$

$$N_{IAI} = \frac{0.5(q_{10}+q_{20}-2q_{10}q_{20})^2\phi_A}{q_{10}+q_{20}-2q_{10}q_{20}\phi_A} \quad \text{(s24d)}$$

AA-type MBP cannot initiate copolymerization since it does not contain functional groups. BFI and IA- and IAI-type MBP can further initiate polymerization because they can generate mono- and biradicals upon further initiation, while BFI only produces MBPs of monomer B.

Let the extent of initiation of the second polymerization q_3 be unity, or all residual I are initiated.

To estimate the $DP_{n,co}$ of final product, we need to calculate the number of total terminal groups.

The terminal group A is derived from the product of the first polymerization and its number is given by Eqn. 25a according to Eqn. s8a.

$$N_A = (q_{10} + q_{20})\overline{\phi_A} = 2q_0\overline{\phi_A} \quad \text{(s25a)}$$

The terminal group B is formed by non-coupling reaction of radical B in the second polymerization. When BFI is completely initiated, the number of radicals formed in the second polymerization is equal to the number of I. The number of residual I at the beginning of the second polymerization (N_I')is given by

$$N_I' = (2 - q_{10} - q_{20}) \quad \text{(s25b)}$$

Let the termination factor of radical B be ϕ_B. The number of terminal group B generated by non-coupling reaction of radical B (N_B) is given by

$$N_B = N_I'\overline{\phi_B} = (2 - q_{10} - q_{20})\overline{\phi_B} = 2(1 - q_0)\overline{\phi_B} \quad \text{(s25c)}$$

The number of total terminal groups is the sum of A and B groups. If BFI is completely initiated, the $DP_{n,co}$ of the total polymers in terms of X is given by

$$DP_{n,co} = \frac{1}{0.5(N_A+N_B)} = \frac{2}{2\overline{\phi_B}+2q_0(\phi_B-\phi_A)} = \frac{1}{\overline{\phi_B}+q_0(\phi_B-\phi_A)} \quad \text{(s26)}$$

When $\phi_A = \phi_B$ and $q_1 = q_2 = q$, the Eqn. s26 and s17m are the same if q =1.

1.11 Summary

Some important parameters of *s*- and *c*-initiation cases obtained by theoretical analysis of LRAsCP are summarized in **Table S1**.

2. Experimental Section

2.1 Materials

Styrene, methyl methacrylate (MMA) and butyl methacrylate (BMA) were purified by normal method and stored under -5 °C. Tetrahydrofuran (THF) were purified by normal method before use. Benzyl bromide (98%, J&K), α, α'-dibromo-p-xylene (98%, J&K), 2-bromoisobutyric acid (>98.0%, TCI), dimanganese decacarbonyl (98%, STREM) were used as received without purification.

2.2 Characterization

Number-average molecular weight (M_n) and molecular weight distributions (Đ) were determined by gel permeation chromatograph (GPC) on a Agilent-1260 Infinity II equipped with two PL gel 10 μm MIXED-B columns against linear PS/PMMA standards and THF as the eluent at a flow rate of 1.0 mL/min at 40 °C. ^{1}H (400 MHz) and ^{13}C (100 MHz) NMR spectra were recorded at room temperature by a Bruker spectrometer using tetramethylsilane (TMS) as the internal standard and $CDCl_3$ as the solvent. High-resolution mass spectra (HRMS) were performed on Agilent 7250 GC/QTOF mass spectrometer.

2.3 Synthesis of initiators

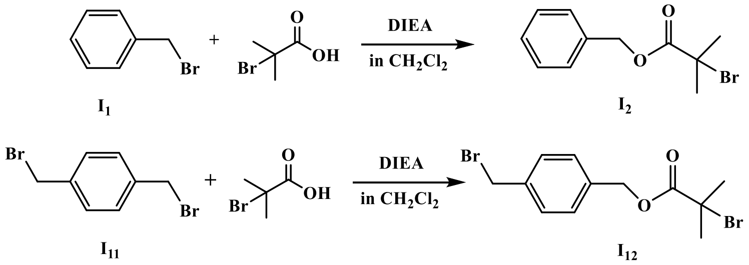

Scheme S3 Preparation of initiators

Four initiators were used in polymerization. Benzyl bromide (**I_1**) and α, α'-dibromo-p-xylene (**I_{11}**) were used as received. Benzyl 2-bromo-2-methylpropanoate (**I_2**) and 4-(bromomethyl) benzyl 2-bromo-2-methylpropanoate (**I_{12}**) shown in **Scheme S2** were prepared by the same method using **I_1** and **I_{11}** and 2-bromoisobutyric acid as reagents by published method.[1]

251 mg (1.5 mmol) of 2-bromoisobutyric acid (BiBA) and 119 μL (1.5 mmol) of benzyl bromide were dissolved in 30 mL of DCM at 25 °C in a flask. 120 μL (0.75 mmol) of N, N-diisopropylethylamine (DIEA) was injected into the flask and the mixture was kept at 25 °C. After 3 hours, 120 μL (0.75 mmol) of DIEA was added to the mixture. After 4.5 hours, the reaction was stopped and the mixture was diluted with 25 mL of DCM, washed with deionized water (3×30 mL) and 30 mL of saturated saline solution, and dried with anhydrous magnesium sulfate. After filtration, the solution was concentrated and dried under vacuum. The compound was purified by passing through a 300-400 mesh silica gel column (PE: EA=50:1(v/v)). 153 mg of a light-yellow oil was obtained after drying (yield 59 %).

Benzyl 2-bromo-2-methylpropanoate (**I$_2$**) ^{1}H-NMR (400 MHz, $CDCl_3$, ppm): δ = 7.38 (m, 5H), 5.21 (s, 2H), 1.95 (s, 6H). ^{13}C-NMR (101 MHz, $CDCl_3$, ppm): δ = 171.61, 135.50, 128.69, 128.43, 127.99, 67.67, 55.78, 30.87. GC-QTOF-HRMS (EI, m/z): calcd. for $C_{11}H_{13}O_2$ $[M\text{-}Br]^+$: 177.0910, found : 177.0906.

(Bromomethyl)benzyl 2-bromo-2-methylpropanoate (**I$_{12}$**) was prepared by the same method as **I$_2$**. The compound was eluted by PE : EA = 50: 1(v/v). The pale-yellow oil was obtained (yield 28 %).

(Bromomethyl)benzyl 2-bromo-2-methylpropanoate (**I$_{12}$**) ^{1}H-NMR (400 MHz, $CDCl_3$, ppm): δ = 7.37 (m, 4H), 5.20 (s, 2H), 4.50 (s, 2H), 1.95 (s, 6H). ^{13}C-NMR (101 MHz, $CDCl_3$, ppm): δ = 171.45, 137.89, 135.68, 129.30, 128.28, 67.04, 55.57, 32.99, 30.74. GC-QTOF-HRMS (EI, m/z): calcd. for $C_{12}H_{15}Br_2O_2$ $[M+H]^+$: 348.9439, found : 348.9436.

2.4 Polymerization procedure

2.4.1 Homopolymerization initiated by BFI

For example, **I$_{11}$** and $Mn_2(CO)_{10}$ were degassed and backfilled with nitrogen three times in a Schlenk flask under light shielding condition. A mixed oxygen- free solution of styrene and THF were added. Immediately after mixing, the flask was immersed in thermostatic water bath at 50 °C under the 8 W visible light. The polymerization was stopped by diluted with 2 mL THF and precipitated by cold methanol at certain times. The product was obtained by filtration and vacuum drying at 60°C. The conversion of polymer was determined by gravimetry. The polymer was characterized by GPC.

2.4.2 Copolymerization initiated by BFI

(1) In-situ addition of the comonomer

4 mL scale of LRAsCP of styrene of under the same polymerization conditions and feed ratio described in Table S1 was carried out. After 4 h, the polymerization was suspended by turning off the light. 2 mL of mixture was sampled and treated later. 500 μL (4.48 mmol) of MMA was added to the residual polymerization media and the polymerization was continued by turning on the light. After 190 min, the polymerization was stopped by diluted with 2 mL of THF and precipitation by cold methanol. The product was obtained by centrifugation and drying under vacuum at 60 °C. 371 mg of MBcP-1 was obtained and the conversion was 19%. The resultant polymer was characterized by ^{1}H-NMR and GPC.

On the other hand, 2 mL of sampled solution was precipitated by methanol and resultant polymer was dried under vacuum. 199 mg of MBP-1 was obtained and the monomer conversion of the first step polymerization was 30%. It was characterized by GPC and M_n of 10 KDa and Đ of 1.7 were obtained. The obtained polymer was termed macro-BFI.

According to the result of sampling, the initial feed ratio of the second-step polymerization was [St] : [MMA] = 1:1 (mol/mol). The conversion of the second step polymerization can be calculated and the data was given in above paragraph.

(2) Initiation using macro-BFI

MBP-1 (110 mg) and $Mn_2(CO)_{10}$ (4 mg, 0.010 mmol) were dissolved in 1.730 mL THF. The mixed monomers of BMA (125 μL 0.78 mmol) and St (80 μL, 0.78 mmol) ([St]:[BMA] = 1:1, mol/mol) were added. The flask was immersed in thermostatic water bath at 50 °C under the 8 W visible light. After 4 hours, the polymerization was stopped by dilution with 2 mL of THF and precipitation by cold methanol. The product was obtained by centrifugation and drying under vacuum at 60 °C. 146 mg of MBcP-2 was obtained and the monomer conversion was 19%. The polymer was characterized by ^{1}H-NMR and GPC.

2.5 Cleavage of MBP

The MBP prepared by **I$_{12}$** can be cleaved. The sample 7 in **Table S3** was dissolved in 30 mL THF at 60 °C. 15 mL of 1 M CH_3OH solution of NaOH was added in portions during 30 min and further

reacted for 16 h. 15 mL of 1 M HCl solution was added to neutralize the reaction mixture and extracted with dichloromethane (20 mL). The organic layer was washed successively with saturated $NaHCO_3$ solution (3×50 mL), deionized water (3×50 mL) and saturated NaCl solution. After dried with anhydrous $MgSO_4$, the solvent was removed under the vacuum, and the product was dried under the vacuum at 60 °C. The number-average molecular weight and molecular weight distribution of cleaved product was measured by GPC.

Reference

1. Gajanan K. D.; Pedro B. C.; Matthew C. T. H. Synthesis of Novel Peptide Linkers: Simultaneous Cyclization and Labeling. *Organic letters*. **2009**, *11* (20), 4708-4711.

3. Tables

Table S1 The parameters of various MBPs[a]

Parameters	Type of MBP	***s*-Initiation**	***c*-Initiation**	
		$q_1 = q_2 = q$	$q \leq 0.5$	$q > 0.5$
Number of MBP	N_{AA}	$\frac{q^2\overline{\phi_A}^2}{1-q\phi_A}$	0	$\frac{q(2q-1)\overline{\phi_A}^2}{q+\phi_A-2q\phi_A}$
	N_{I_1A}	$\frac{2q\bar{q}\overline{\phi_A}}{1-q\phi_A}$	0	0
	N_{I_2A}		$2q\overline{\phi_A}$	$\frac{2q(1-q)\overline{\phi_A}}{q+\phi_A-2q\phi_A}$
	$N_{I_1AI_1}$		0	0
	$N_{I_1AI_2}$	$\frac{q\bar{q}^2\phi_A}{1-q\phi_A}$	0	0
	$N_{I_2AI_2}$		$q\phi_A$	$\frac{\bar{q}^2\phi_A}{q+\phi_A-2q\phi_A}$
	N_{P_A}	$q(2-\phi_A-q)$	$q(2-\phi_A)$	$1-q\phi_A$
Fraction of MB	F_{AA}	$\frac{q\overline{\phi_A}^2}{(1-q\phi_A)(2-\phi_A-q)}$	0	$\frac{q(2q-1)\overline{\phi_A}^2}{(q+\phi_A-2q\phi_A)(1-q\phi_A)}$
	F_{IA}	$\frac{2(1-q)\overline{\phi_A}}{(1-q\phi_A)(2-\phi_A-q)}$	$\frac{\overline{\phi_A}}{1-0.5\phi_A}$	$\frac{2q(1-q)\overline{\phi_A}}{(q+\phi_A-2q\phi_A)(1-q\phi_A)}$
	F_{IAI}	$\frac{(1-q)^2\phi_A}{(1-q\phi_A)(2-\phi_A-q)}$	$\frac{0.5\phi_A}{1-0.5\phi_A}$	$\frac{(1-q)^2\phi_A}{(q+\phi_A-2q\phi_A)(1-q\phi_A)}$
	W_{AA}	$\frac{q^2\overline{\phi_A}^2}{(1-q\phi_A)^2}$	0	$\frac{(2q-1)q^2\overline{\phi_A}^2}{(q+\phi_A-2q\phi_A)^2}$
DP_n of MBP	$DP_{n,AA}$	$\frac{1}{1-q\phi_A}$	-	$\frac{q}{q+\phi_A-2q\phi_A}$
	$DP_{n,IA}$	$\frac{1}{1-q\phi_A}$	1	$\frac{q}{q+\phi_A-2q\phi_A}$
	$DP_{n,IAI}$	$\frac{2-q\phi_A}{1-q\phi_A}$	2	$\frac{2q+\phi_A-2q\phi_A}{q+\phi_A-2q\phi_A}$
	DP_n	$\frac{2-q}{2-q-\phi_A}$	$\frac{1}{1-0.5\phi_A}$	$\frac{1}{1-q\phi_A}$
	$DP_{n,GPC}$	$\frac{N_{K,GPC}}{2-\phi_A}+\frac{1-q}{2-\phi_A-q}$	$\frac{N_{K,GPC}}{2-\phi_A}+\frac{0.5}{1-0.5\phi_A}$	$\frac{N_{K,GPC}}{2-\phi_A}+\frac{1-q}{1-q\phi_A}$
M_n of MBP [b)]	total	$\frac{M_{n,0}}{q(2-q-\phi_A)}$	$\frac{M_{n,0}}{q(2-\phi_A)}$	$\frac{M_{n,0}}{1-q\phi_A}$
N_K of MBP	total	$\frac{2-\phi_A}{2-\phi_A-q}$	1	$\frac{q(2-\phi_A)}{1-q\phi_A}$
f of MBP	total	$\frac{2(1-q)}{(2-\phi_A-q)}$	$\frac{1}{1-0.5\phi_A}$	$\frac{2(1-q)}{1-q\phi_A}$
q [c)]		$\frac{(2-\phi_A)\pm\sqrt{(2-\phi_A)^2-4R}}{2}$	$\frac{R}{(2-\phi_A)}$	$\frac{1-R}{\phi_A}$

a) $\overline{q_1} = 1 - q_1$, $\overline{q_2} = 1 - q_2$, $\overline{\phi_A} = 1 - \phi_A$; b) $M_{n,0} = \frac{A_0 Cm_A}{X_0}$. c) $R = \frac{M_{n,0}}{M_n}$

Table S2 LRAsCP of styrene initiated by BFI I_{11} and I_1[a]

Run	Time (h)	Conversion (wt %)	M_n/Đ[b] (KDa/-)	q[c]	$DP_{n,GPC}$[d]	DP_n[e]	$N_{K,GPC}$[f]
1	1	8	4.5/1.8	0.16	2.1	2.0	1.2
2	2	16	5.2/2.0	0.36	2.2	2.3	1.4
3	3	24	7.2/2.2	0.44	2.7	2.5	1.9
4	4	32	10.0/2.6	0.68	3.3	3.4	2.7
5	5	37	15.0/3.3	0.82	4.5	4.7	4.1
6	6	42	20.7/5.2	0.88	5.9	5.9	5.6
7	7	49	28.6/8.9	0.91	7.8	6.8	7.7
8[g]	2	14	3.7/1.7	-	-	1.9	1

a) Reaction conditions: $[I]_0$ = 0.08 M, $[St]_0$: $[I]_0$: $[Mn_2(CO)_{10}]_0$ = 40: 1: 0.52, 50 °C, in 2 mL THF, light power: 8 W, distance: 10 cm. b) The number-average molecular weight (M_n) and polydispersity (Đ) of polymer measured by GPC. c) The extent of initiation calculated by Eqn. s18b. d) The number-average degree of polymerization estimated by Eqn. s17n. e) The number-average degree of polymerization estimated by Eqn. s17m. f) Calculated by Eqn. s15. g) The initiator is monofunctional benzyl bromide I_1.

Table S3 LRAsCP of styrene initiated by BFI I_{12} and I_2[a]

Run	Time (h)	Conversion (wt %)	M_n/Đ[b] (KDa/-)	$M_{n,c}$/$Đ_c$[c] (KDa/-)
1	1	9	5.5/1.5	-
2	2	14	6.5/1.6	-
3	3	22	7.5/1.7	-
4	4	30	8.7/1.9	-
5	5	36	11.1/2.1	-
6	6	40	17.5/3.9	-
7	7	48	21.6/7.4	5.1/1.7
8[d]	2	14	4.6/1.6	-

a) Reaction conditions: $[I]_0$ = 0.08 M, $[St]_0$: $[I]_0$: $[Mn_2(CO)_{10}]_0$ = 40: 1: 0.52, 50 °C, in 2 mL THF, light power: 8 W, distance: 10 cm. b) The number-average molecular weight (M_n) and polydispersity (Đ) of polymer measured by GPC. c) The number-average molecular weight ($M_{n,c}$) and polydispersity ($Đ_c$) of cleaved polymer measured by GPC. d) The initiator is monofunctional I_2.

Table S4 Two-step LRAsCP initiated by I_{11}

Step	Sample	Time (h)	Conv. (wt %)	f_{st} [a]	M_n/Đ[b] (KDa/-)	$F_{st,1}$[c] (mol %)	$F_{st,2}$[d] (mol %)	W[e] (wt %)	T_g[f] (°C)
1[g]	MBP-1	4	30	1.0	9.4/2.0	100	100	0	97
2[h]	MBcP-1	3.2	33	0.5	34.7/5.1	81	59	46	103
2[i]	MBcP-2	4	19	0.5	38.6/5.0	92	64	33	53

a) Feed ratio of monomer composition. b) The number-average molecular weight (M_n) and polydispersity (Đ) of polymer measured by GPC. c) Polymer composition of MBP. d) Polymer composition of blocks produced in the second step polymerization. e) The weight percentage of copolymer block. f) Glass transition temperature of MBP. g) Reaction conditions of first step polymerization: $[I]_0$ = 0.08 M, $[St]_0 : [I]_0 : [Mn_2(CO)_{10}]_0$ = 40 : 1: 0.52, 50 °C, in 4 mL THF, light power: 8 W, distance: 10 cm. h) In-situ second LRAsCP of styrene and MMA. i) The second LRAsCP of styrene and BMA using MBP-1 as macro-initiator.

Table S5 LRAsCP of various monomers initiated by $\mathbf{I_{11}}$ and $\mathbf{I_1}$[a]

**Monomer**	**Conv. (I_1) (wt %)**	**M_n' (I_1)[b] (KDa)**	**Conv. (I_{11}) (wt %)**	**M_n (I_{11})[c] (KDa)**	**$N_{K,GPC}$[d]**	**ϕ[e]**	**q[f]**
Styrene	24	4.7	24	8.7	1.85	0.93	0.49
MMA	49	17.2	50	24.4	1.42	0.33	0.49
BMA	57	26.7	73	34.2	1.28	-0.25	0.49

a) Reaction conditions: $[I]_0$ = 0.08 M, $[St]_0$: $[I]_0$: $[Mn_2(CO)_{10}]_0$ = 40: 1: 0.52, 50 °C, 3 h, in 2 mL THF, light power: 8 W, distance: 10 cm. b) The number-average molecular weight of polymer prepared by $\mathbf{I_1}$ measured by GPC. c) The number-average molecular weight of polymer prepared by $\mathbf{I_{11}}$ measured by GPC. d) The ratio of M_n/M_n'. e) Terminal factor of monomer and the value of styrene is 0.93. f) Calculation based on Eqn. 14.

4. Collection of spectra

4.1 NMR spectra

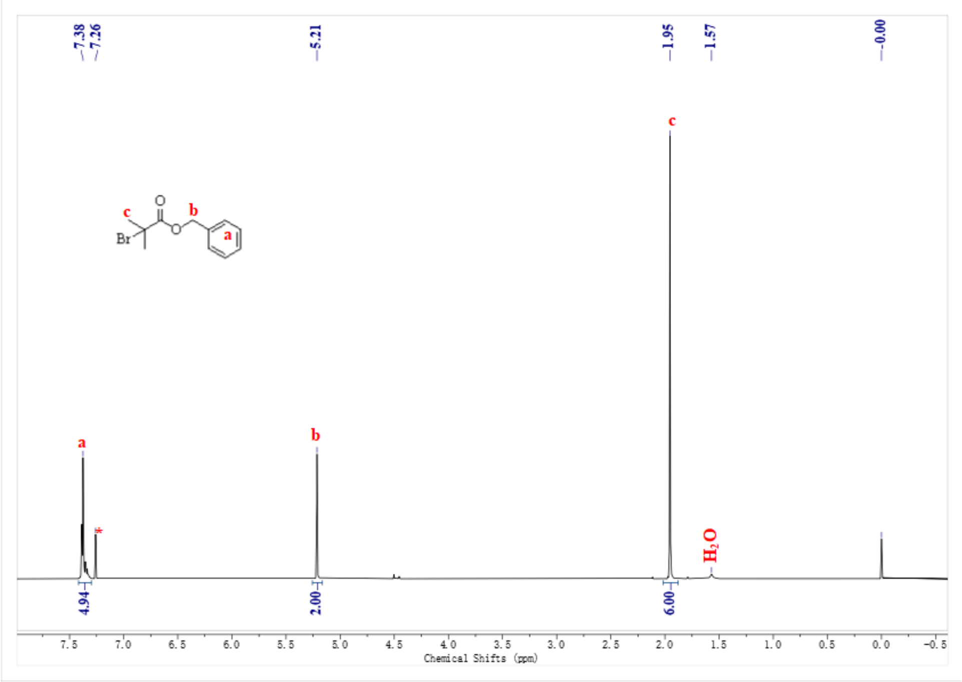

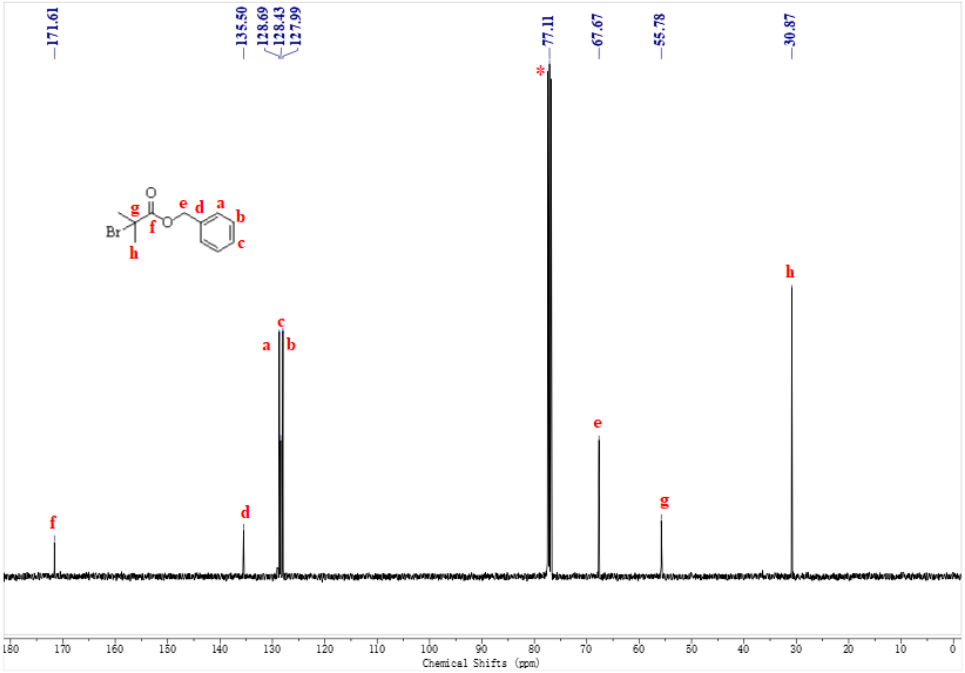

Figure S1 ^{1}H-NMR (400 MHz) and ^{13}C-NMR (100 MHz) spectra of **I_2** (*$CDCl_3$)

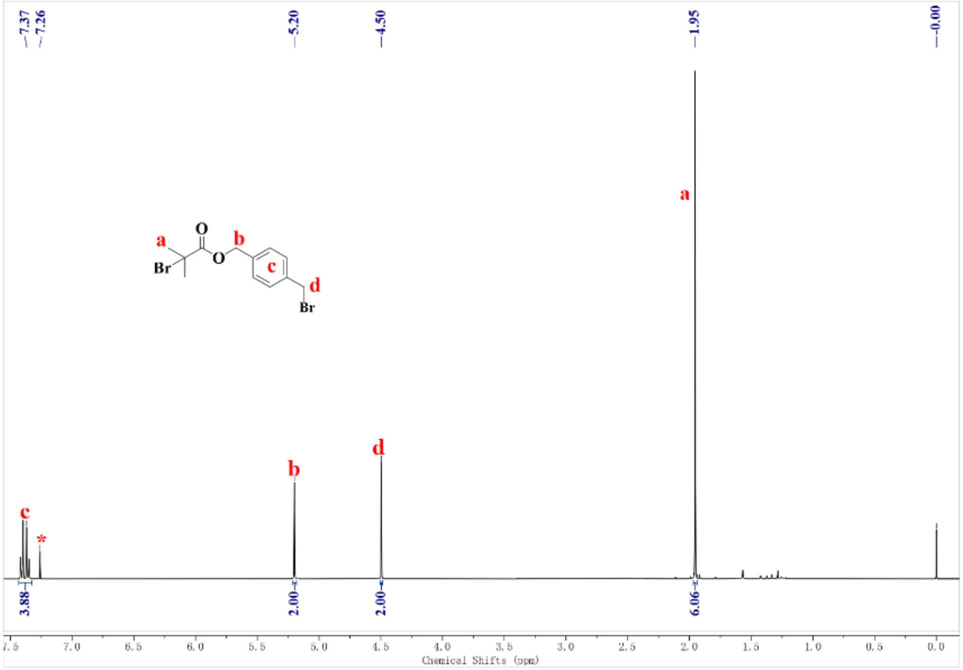

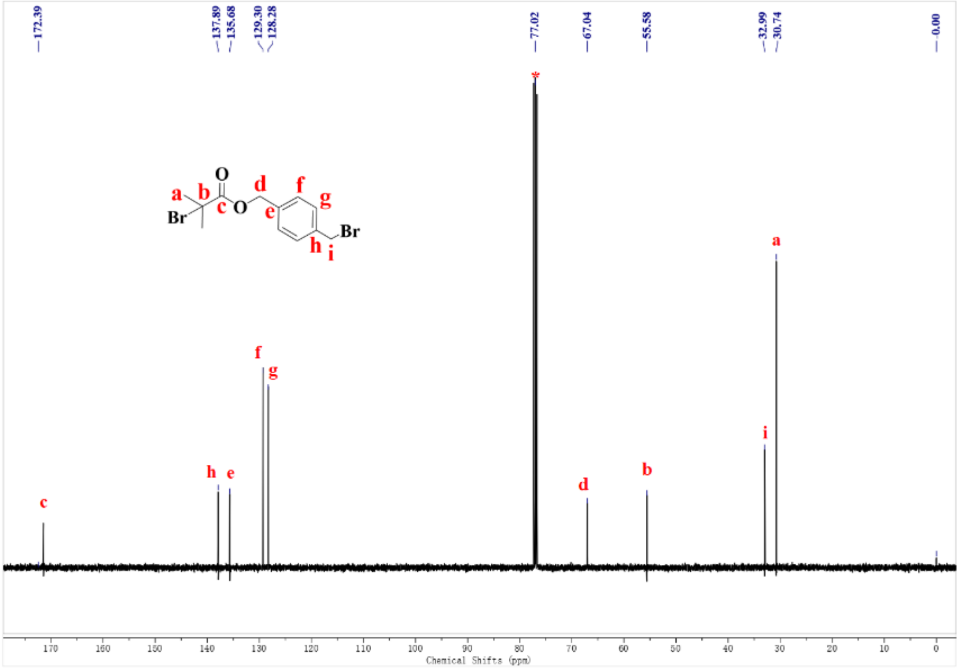

Figure S2 ^{1}H-NMR (400 MHz) and ^{13}C-NMR (100 MHz) spectra of $\mathbf{I_{12}}$ (*$CDCl_3$)

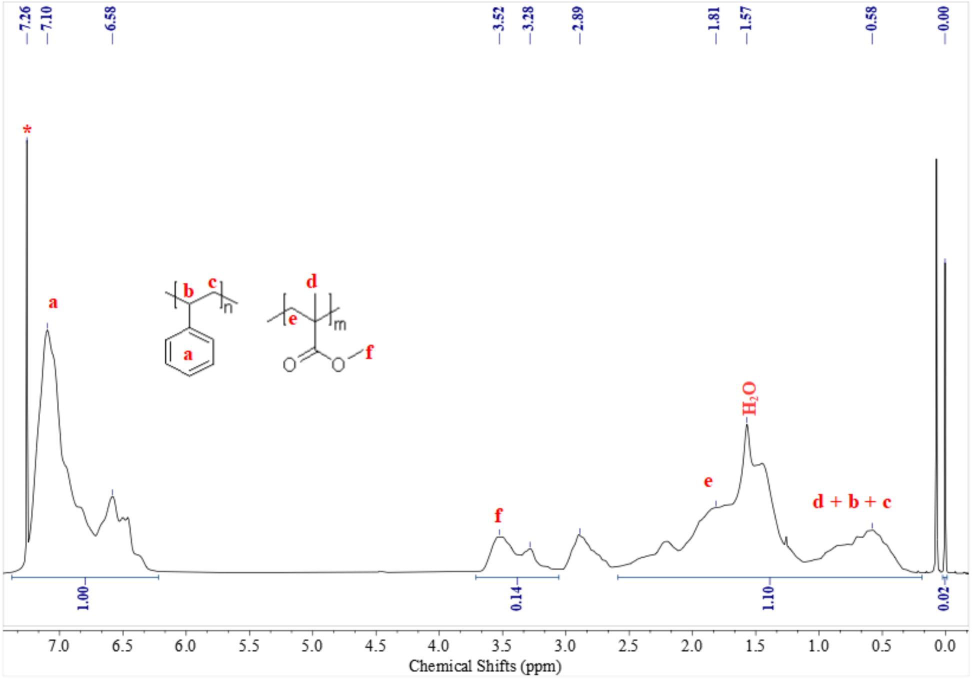

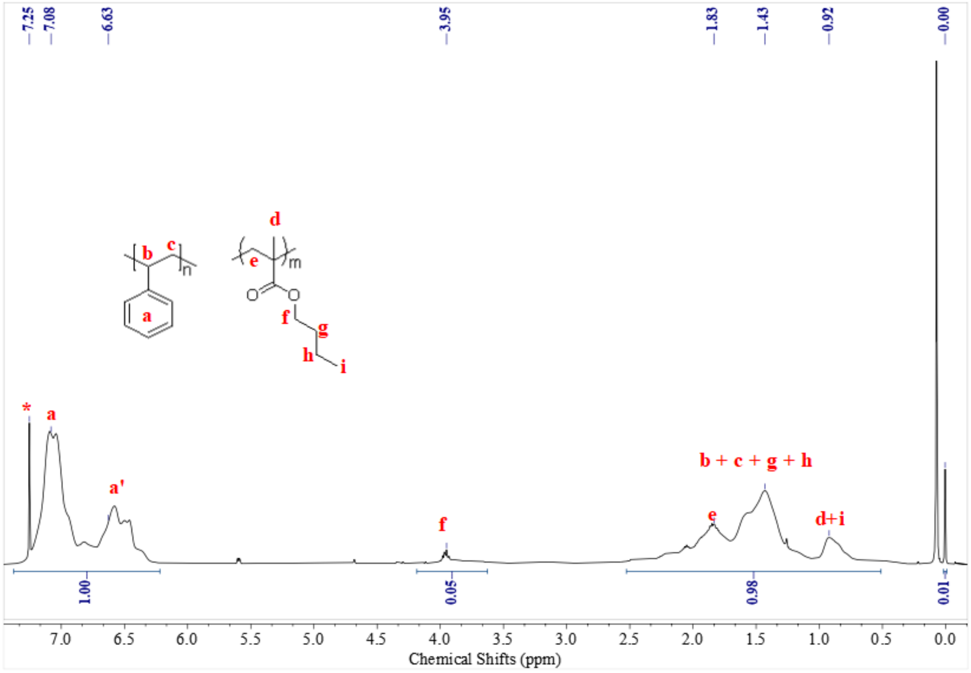

Figure S3 ^{1}H-NMR (400 MHz) spectra of MBcP-1 (top) and MBcP-2(bottom) (*$CDCl_3$)

4.3 GPC curves of MBPs

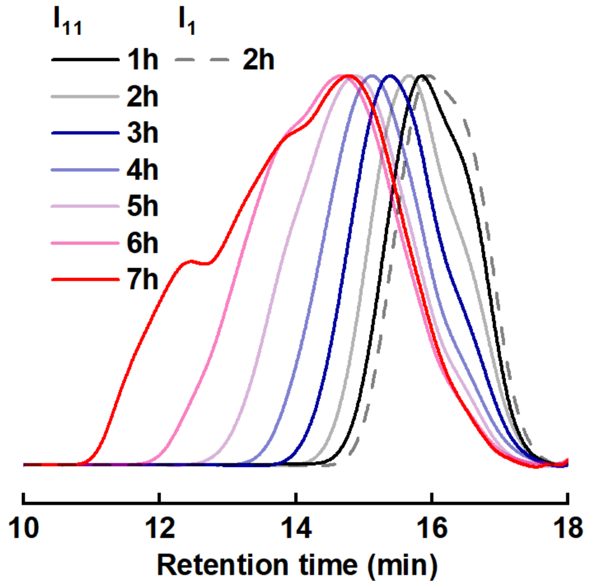

Figure S4 GPC curves of polystyrene prepared by I_{11} and I_1 at various times

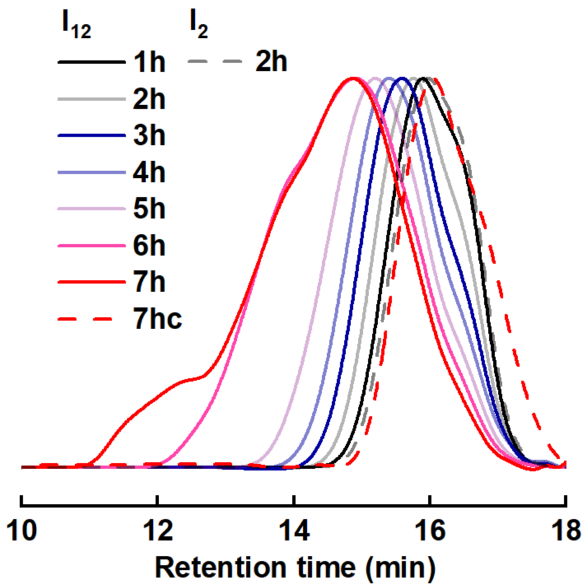

Figure S5 GPC curves of polystyrene prepared by I_{12} and I_2 at various times
